# Biomechanical Markers of Forward Hop-Landing After ACL-Reconstruction: A Pattern Recognition Approach

**DOI:** 10.1007/s10439-022-02921-4

**Published:** 2022-01-31

**Authors:** Prasanna Sritharan, Mario A. Muñoz, Peter Pivonka, Adam L. Bryant, Hossein Mokhtarzadeh, Luke G. Perraton

**Affiliations:** 1 La Trobe Sports and Exercise Medicine Research Centre, La Trobe University, Bundoora, Australia; 2 School of Mathematics & Statistics, University of Melbourne, Melbourne, Australia; 3 School of Mechanical, Medical & Process Engineering, Queensland University of Technology, Brisbane, Australia; 4 Centre for Health, Exercise and Sports Medicine, University of Melbourne, Melbourne, Australia; 5 Department of Mechanical Engineering, University of Melbourne, Melbourne, Australia; 6 Department of Physiotherapy, Monash University, Melbourne, Australia

**Keywords:** Anterior cruciate ligament, Feature selection, Principal component analysis, Knee osteoarthritis, Musculoskeletal modelling, Machine learning

## Abstract

Biomechanical changes after anterior cruciate ligament reconstruction (ACLR) may be detrimental to long-term knee-joint health. We used pattern recognition to characterise biomechanical differences during the landing phase of a single-leg forward hop after ACLR. Experimental data from 66 individuals 12-24 months post-ACLR (28.2 ± 6.3 years) and 32 controls (25.2 ± 4.8 years old) were input into a musculoskeletal modelling pipeline to calculate joint angles, joint moments and muscle forces. These waveforms were transformed into principal components (features), and input into a pattern recognition pipeline, which found 10 main distinguishing features (and 8 associated features) between ACLR and control landing biomechanics at significance $\alpha =0.05$. Our process identified known biomechanical characteristics post-ACLR: smaller knee flexion angle; less knee extensor moment; lower vasti, rectus femoris and hamstrings forces. Importantly, we found more novel and less well-understood adaptations: smaller ankle plantar flexor moment; lower soleus forces; and altered patterns of knee rotation angle, hip rotator moment and knee abduction moment. Crucially, we identified, with high certainty, subtle aberrations indicating landing instability in the ACLR group for: knee flexion and internal rotation angles and moments; hip rotation angles and moments; and lumbar rotator and bending moments. Our findings may benefit rehabilitation and assessment for return-to-sport 12–24 months post-ACLR.

**Supplementary Information:**

The online version contains supplementary material available at 10.1007/s10439-022-02921-4.

## Introduction

The single-leg hop-for-distance is routinely used in the evaluation of individuals after surgery to reconstruct a ruptured anterior cruciate ligament^26,31^ (ACL). During the landing phase, the individual must arrest the forward motion of the body, while supporting the body against gravity.^38^ Due to the dynamic nature of the task, and the consequent neuromuscular demands at the knee, the landing phase of single-leg hop-for-landing is known to stress the ACL^1^. During single-leg landing tasks, ACL-reconstructed (ACLR) individuals have shown altered biomechanics at the hip, knee and ankle^12,15,25,27,45^ when landing on the involved knee. In particular, ACLR individuals have demonstrated smaller peak knee flexion angles^20,27,28,38,45^ and smaller peak knee extensor moments^12,20,27,28,38^ compared to controls and/or the uninjured limb. However, these findings are based on discrete data points in the landing phase determined *a priori*, and do not take into account the whole temporal waveforms of these biomechanical variables.^4^

Computer-based musculoskeletal modelling is a powerful, non-invasive approach^29^ that can facilitate an estimation of altered biomechanical control strategies after ACLR for many types of movements. However, the data resulting from these analyses is multidimensional and demonstrates a non-linear relationship with quasi-periodic temporal dependence.^3,8^ Furthermore, the data exhibits variability, even within subjects and sessions^3,6^. This variability can be attributed to measurement error (e.g. inconsistent marker alignment and instrumentation^3,6^) and/or the individuals’ neuromuscular adaptations to their impairments^6^. The latter often results in functionally optimal, although abnormal, movement patterns. Movement variability complicates the objective examination of the data. In this regard, pattern recognition techniques may be suitable for the examination of biomechanical waveform data with such inherent variability.

Principal Component Analysis (PCA) is one of the most commonly used approaches for analysing variance in multivariate data, and can be applied to the analysis of biomechanical temporal waveform data using pattern recognition methods.^6^ PCA can reveal clinically relevant information that would otherwise be difficult to interpret from the original waveforms in healthy^5,16,34,44^ and pathological^8,18,21,23,33,35^ movement patterns. PCA attempts to find a smaller set of new, non-redundant features, known as principal components, which sufficiently capture the observed total variation in the original variables^3^. It is unbiased and it does not require an *a priori* determination of features to extract.^5^ Although principal components are mathematically-abstract features, meaningful interpretation of the principal components can be undertaken with expert knowledge of human motion.^3^ Often, PCA is followed by statistical hypothesis testing,^5,8,44^ classification^2,16^ or regression models^21^ to identify those features that are good predictors of the presence of anomalous movement patterns.

Despite the widespread use of PCA in biomechanical studies, only two studies have used PCA to investigate differences in biomechanics between ACLR individuals and controls during dynamic tasks. Both Leporace *et al*.^21^ and Sanford *et al*.^35^ investigated walking gait post-ACLR using PCA and post-hoc classification methods; however, these studies only examined biomechanical variables associated with the knee. Leporace *et al*.^21^ examined only knee-joint angles, finding that aberrations in knee internal rotation and knee adduction angles during gait were more important than knee flexion angle in distinguishing between ACLR and controls. Sanford *et al*.^35^ additionally found that the knee adduction moment, which was elevated in early and late stance, was also a key variable in distinguishing between the groups. However, neither study included variables at the trunk, hip or ankle, and did not consider muscle forces. To our knowledge, no study using pattern recognition methods has examined a demanding dynamic task that is known to stress the ACL, such as the single-leg hop-for-distance.

Understanding the key distinguishing features of single-leg hop-for-distance may help improve the clinical assessment of individuals post-ACLR, and help mitigate re-injury or future osteoarthritis. Furthermore, it may, in future, facilitate the automated machine-based evaluation of individuals post-ACLR by interrogating subtle or hidden features of landing biomechanics that may be difficult to observe directly. Therefore, the aim of this study was to apply musculoskeletal modelling and pattern recognition methods, to find a minimum set of features of muscle forces, joint angles and joint moments, that could distinguish between the biomechanics of ACLR individuals and uninjured controls during a single-leg forward hop-landing task. As lower peak knee flexion angle and knee extensor moment are distinct identifying characteristics of single-leg landing tasks post-ACLR^38^, we hypothesised that principal components associated with these respective variables would be included in the set of selected features.

## Materials and Methods

### Data Collection and Musculoskeletal Modelling

Sixty-six participants (28.2 ± 6.3 years, 24 (36%) women, height: 1.75 ± 0.10 m, mass: 78.3 ± 14.8 kg, 17 ± 3 months after ACLR) from a previously-described cohort of 111 eligible patients with unilateral single-bundle ACLR using a semitendinosis-gracilis tendon graft.^31^ and 32 uninjured control subjects (25.2 ± 4.8 years old, 17 (47%) women, height: 1.70 ± 0.08 m, mass: 68.0 ± 11.4 kg) participated in this study after providing informed consent. Ethical approval (ID: 1136167) for the study was provided by the Behavioural and Social Sciences Human Ethics sub-committee at the University of Melbourne. Surgery was performed by one of two experienced orthopaedic surgeons using identical surgical techniques. Inclusion criteria were a successful unilateral ACLR as determined by clinical examination by the orthopaedic surgeon. Subjects with self-reported knee instability, knee instability on clinical examination, revision ACLR, other surgery since ACLR or any other conditions affecting walking, sports activity or daily function were excluded.

Data collection was undertaken at the Movement Research Laboratory, Centre for Health, Exercise and Sports Medicine, University of Melbourne. Participants completed an anticipated single-limb forward hop. The experimental procedure has been previously detailed,^38^ and only briefly described below. Hop distance was normalised to 100% of leg length (greater trochanter to floor), with white tape used to mark the subject-specific take-off point on the floor and the landing point in the centre of the force plate. Participants took three steps forward using their preferred step length, took off at the marked take-off point, and landed on the cross in the centre of the force plate with the same leg with arms folded across their chest. ACLR participants used their affected leg while controls performed the task on their right leg. Landings were deemed successful if participants were able to maintain balance for five seconds after landing without moving their arms. Spatial marker trajectories were recorded using a 14-camera Vicon motion analysis system (Oxford Metrics, Oxford, UK) at 120 Hz and ground force data were recorded using a single force plate (AMTI Watertown, MA, USA).

Modelling and analyses were performed using OpenSim 4.1^7^ *via* the API using MATLAB R2020b (MathWorks Inc. Natick, MA, USA). For each participant, a musculoskeletal model was generated by scaling a generic 27-degree-of-freedom 92-muscle model. The ankles and subtalar-joints were modelled as pin-joints, while the hips and knees were modelled as a ball-joints. The metatarsophalangeal-joints were modelled as pin-joints, but locked at their reference positions. The head, arms and torso were merged into a single body which articulated with the pelvis *via* the back-joint, modelled as a ball-joint.

For each participant and trial, joint angles were calculated using an inverse kinematics analysis by minimising the distance between model and experimental marker trajectories.^24^ Joint moments were calculated using inverse dynamics by applying the joint kinematics and measured ground forces to the model. Computed Muscle Control^41^ (CMC) was then used to calculate muscle forces. The internal knee adduction and rotation moments were excluded from CMC. Prior to calculation of muscles forces, the model’s torso centre-of-mass and trial joint kinematics were adjusted using the Residual Reduction Algorithm (RRA) to minimise dynamic inconsistencies. All analyses were performed for the *landing phase* of the task, defined as the period from initial foot strike to maximum knee flexion angle.

### Pattern Recognition Pipeline

The temporal waveforms of 30 biomechanical variables (13 joint angles, 10 joint moments and 7 muscle forces) for each of 452 recorded trials from all subjects (301 ACLR trials; 151 control trials) were input into a pattern recognition pipeline, implemented in MATLAB, that found a minimal set of features, called principal components, that could best differentiate between the biomechanics of ACLR and control groups.

### Principal Component Analysis

For each variable, the individual trial waveform data were first transformed into a set of features called principal components that explain the maximum amount of variance in the original variables in a process known as Principal Component Analysis (PCA). This process is described in detail by Wrigley *et al*.,^44^ therefore we present only a summary here. PCA uses an orthogonal transformation that converts the $n\times p$ matrix of waveform data **X** into an $n\times p$ matrix of mutually uncorrelated principal component scores **Z**. There is an independent matrix **X** for each biomechanical variable, e.g. knee adduction angle or rectus femoris muscle force. For each variable’s matrix **X**, *n* rows represent trials, while *p* columns represent individual temporal samples of the waveform. ACLR and control trials are pooled such that $n=452$, and $p=101$, with each temporal sample representing 1% of the landing phase within the 0%-100% range. The principal component scores matrix **Z** for that variable was then calculated by the linear transformation:

1 $$\mathbf{Z}=\mathbf{X}\mathbf{U}$$

where **U** is the $p\times p$ matrix of eigenvectors of **R**, which is the weighted correlation matrix of **X**. Each eigenvector ${\mathbf{u}}_{j}$, where $j\in [1,p]$, in the matrix of eigenvectors **U**, is a set of coefficients that is applied to each row of **X** to produce the matrix of principal scores **Z**. For each given biomechanical variable, every element ${\mathbf{z}}_{ij}$ of its matrix **Z** is known as a *principal component score*, and is a measure of the degree to which the shape of that variable’s waveform for trial *i* corresponds to the shape of the principal component eigenvector ${\mathbf{u}}_{j}$.

All trials from all subjects were input into the PCA, however some participants recorded more trials than others. Thus, a normalized weight was applied to each row $i\in \left[1,n\right]$ in **X** when constructing the weighed correlation matrix **R**. Suppose row *i* represents a trial from participant S, who recorded ${q}_{\mathrm{S}}$ trials in total. We define a unique factor:

2 $${\tau }_{i}=\frac{1}{{q}_{\mathrm{S}}}$$

Therefore, the normalised weight applied to row *i* is:

3 $${w}_{i}=\frac{{\tau }_{i}}{\sum_{m=1}^{n}{\tau }_{m}}$$

#### Parallel Analysis

For each biomechanical variable, the number of principal components was then reduced using Parallel Analysis (PA).^13^ Only those principal components that explained most of the variance in the data are retained, discarding the remainder. That is, if most of the variance in the data is explained by the first *k* principal components, the remaining $p-k$ components were dropped. PA assumes that non-trivial principal components should have eigenvalues larger than principal components derived from random data with the same sample size and number of variables.^10^

To apply PA in the present context, *N* matrixes of size $n\times p$ were generated, whose elements were independent and identically distributed random variables from a standard normal distribution. As larger *N* improves the accuracy of PA, we set $N=1000$. The $n\times n$ correlation matrix was calculated from each random matrix, and its eigenvalues were extracted. The 95th-percentile of each eigenvalue from the random set was estimated and compared with the corresponding eigenvalues of **R**. Principal components with eigenvalues greater than the 95th-percentile were retained. After PA, 108 principal components were retained, a large number that may not have represented the most relevant modes of variation present in the data for the discrimination task.^34^

### Weiss-Indurkhya Independent Features Selection

To further reduce the number of retained features after PA, the Weiss-Indurkhya Independent Features Selection method^43^ was applied to the remaining principal components. For each retained variable, the mean principal component scores were calculated for the ACLR and controls groups, and compared using Welch’s *t*-test. Only features for which the between-group comparison returned $t\ge 2.0$ were retained. For $n=108$, this is approximately a significance level of 95%, i.e. $\alpha =0.05$. Forty-six principal components were retained after this step.

### Sequential Feature Selection

Sequential Feature Selection^14^ (SFS) was then used to obtain the final minimal set of features from those 46 retained thus far. In SFS, the principal components were sequentially added to an empty candidate set until the addition of further features did not improve the stopping criterion or the number of valid features was exceeded. The stopping criterion was defined as the 10-fold cross-validated misclassification rate of a Naïve Bayes classifier, with maximum number of valid features set to 10. The Naïve Bayes Classifier was used because of its simplicity, effectiveness and short training time.^32^ As each iteration of SFS could produce different results due to the random nature of the 10-fold cross validation, 1000 iterations of the SFS were performed, with only the 10 most-frequently selected principal components were retained.

These final 10 principal components were defined as *main features*. Between-group differences in principal component scores for each of the main features were evaluated using Welch’s *t*-tests at a tighter significance level of 99.9% ($\alpha =0.001$), as these features were already significantly different at the 95% level. Effect sizes were calculated using Hedges’ *g*-score.

As principal components are abstract constructs, they require interpretation to have contextual meaning. For each feature, this was accomplished by: comparing the waveforms of the pooled original data that correspond to high and low principal component scores respectively^8,36^; the shape of the eigenvectors, i.e. waveform of the principal component coefficients^36^; and the variance of the original data explained by that feature, i.e., the squared correlation between the temporal samples of the original data and the principal components.^44^

### Associated Features

SFS tends to exclude features that are well-correlated with the main features if they do not increase the accuracy of the model. These *associated features* can aid in interpretation of the results. For each main feature, we calculated the Pearson correlations $\rho$ between it and every other remaining principal component after PA. Associated features were defined as those remaining principal components which correlated moderately ($0.5\le \left|\rho \right|<0.7$) or strongly ($\left|\rho \right|\ge 0.7$) with their respective main feature at significance level $\alpha =0.05$. Between-group differences in principal component scores for each of the associated features were evaluated using *t*-tests at significance level $t\ge 2.0$ for consistency with our Weiss-Indurkhya Independent Features Selection step, noting those which were also significant at $\alpha =0.001$.

## Results

**Figure 1:**
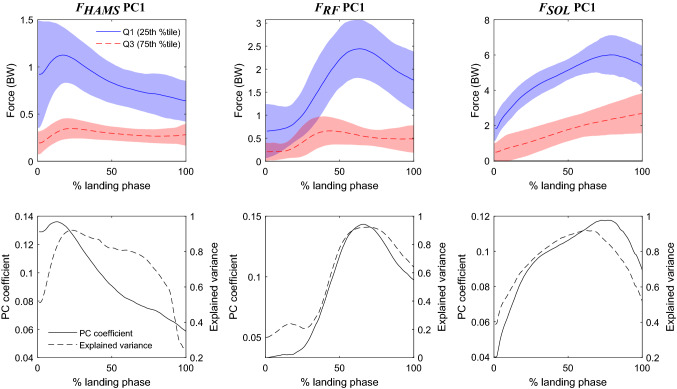
Main features representing muscle forces: *F*_HAMS_ PC1, *F*_RF_ PC1, and *F*_SOL_ PC1. Top row: waveforms of the pooled original data (*F*_HAMS_, *F*_RF_ and *F*_SOL_ respectively), representing the upper (solid blue) and lower (dashed red) quartiles of the principal component scores for each respective feature. Shaded regions represent 1 standard deviation about the respective waveforms. Bottom row: for each feature, waveforms of the principal component coefficients (PC coefficient, solid black) and the squared correlation with the waveforms of the original data (Explained variance, dashed black). Landing phase: from foot strike (0%) to peak knee flexion angle (100%).

**Figure 2:**
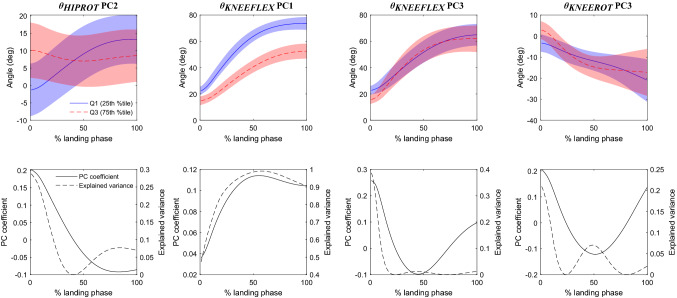
Main features representing joint angles: *θ*_HIPROT_ PC2, *θ*_KNEEFLEX_ PC1, *θ*_KNEEFLEX_ PC3 and *θ*_KNEEROT_ PC3. Top row: waveforms of the pooled original data (*θ*_HIPROT_, *θ*_KNEEFLEX_, *θ*_KNEEFLEX_ and *θ*_KNEEROT_ respectively), representing the upper (solid blue) and lower (dashed red) quartiles of the principal component scores for each respective feature. Shaded regions represent 1 standard deviation about the respective waveforms. Bottom row: for each feature, waveforms of the principal component coefficients (PC coefficient, solid black) and the squared correlation with the waveforms of the original data (Explained variance, dashed black). Landing phase: from foot strike (0%) to peak knee flexion angle (100%).

**Figure 3:**
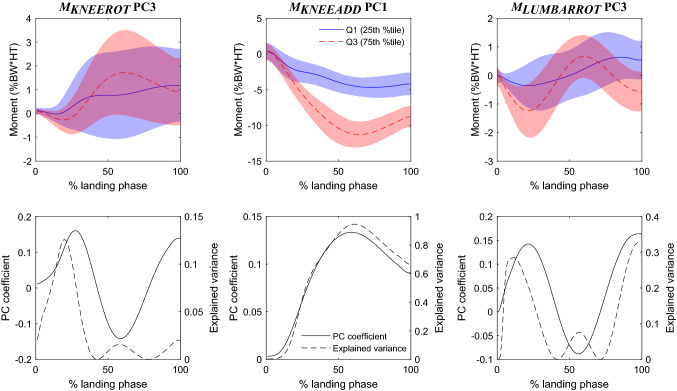
Main features representing joint moments: *M*_KNEEROT_ PC3, *M*_KNEEADD_ PC1, and *M*_LUMBARROT_ PC3. Top row: waveforms of the pooled original data (*M*_KNEEROT_, *M*_KNEEADD_ and *M*_LUMBARROT_ respectively), representing the upper (solid blue) and lower (dashed red) quartiles of the principal component scores for each respective feature. Shaded regions represent 1 standard deviation about the respective waveforms. Bottom row: for each feature, waveforms of the principal component coefficients (PC coefficient, solid black) and the squared correlation with the waveforms of the original data (Explained variance, dashed black). Landing phase: from foot strike (0%) to peak knee flexion angle (100%).

For each variable retained after PA, median of 4 principal components accounting for an average of 98.5% of the total variance was retained (Table 1). This was reduced to 10 main features (Table 2) comprising 3 principal components of muscle forces (Fig. 1), 4 of joint angles (Fig. 2), and 3 of joint moments (Fig. 3). Seven features were able to distinguish between control and ACLR groups at significance level $\alpha =0.001$.

**Table 1:** Proportion of variance explained by each principal component retained after Parallel Analysis for the pooled data, followed by Weiss-Indurkhya independent feature selection and, finally, Sequential Feature Selection.

Variable	Description	Proportion of variance explained (%)						
		PC1	PC2	PC3	PC4	PC5	PC6	Total
Muscle forces								
*F*_GMAX_	Gluteus maximus	76.5	9.4	6.4	4.8			97.2
*F*_GMED_	Gluteus medius	***58.4***	***26.5***	9.9	3.0			97.8
*F*_HAMS_	Hamstrings	***73.2***	15.0	6.3	2.6			97.1
*F*_RF_	Rectus femoris	***68.5***	15.4	8.3	5.1			97.3
*F*_VAS_	Vasti	***64.1***	***17.4***	7.4	***4.0***			92.9
*F*_GAS_	Gastrocnemius	65.3	***20.4***	7.7	3.0			96.5
*F*_SOL_	Soleus	***77.9***	***15.6***	3.5				97.0
Joint angles								
*θ*_HIPFLEX_	Hip flexion	95.0	3.7					98.7
*θ*_HIPADD_	Hip adduction	87.4	***10.2***	2.0				99.6
*θ*_HIPROT_	Hip internal rotation	***89.3***	***8.9***					98.2
*θ*_KNEEFLEX_	Knee flexion	***93.3***	***4.8***	***1.5***				99.7
*θ*_KNEEROT_	Knee internal rotation	***82.9***	13.0	***2.9***				98.8
*θ*_KNEEADD_	Knee adduction	93.4	4.9	***1.4***				99.6
*θ*_ANKLEDF_	Ankle dorsiflexion	51.2	36.5	9.9	***2.1***			99.7
*θ*_PELVISTILT_	Pelvis tilt	95.8	3.5					99.3
*θ*_PELVISLIST_	Pelvis list	86.8	10.1	2.5				99.5
*θ*_PELVISROT_	Pelvis internal rotation	93.0	6.1					99.0
*θ*_LUMBAREXT_	Lumbar extension	***96.5***	2.9					99.4
*θ*_LUMBARBEND_	Lumbar bending	86.1	***10.7***	2.7				99.5
*θ*_LUMBARROT_	Lumbar internal rotation	86.8	***10.2***	2.5				99.5
Joint moments								
*M*_HIPFLEX_	Hip flexor	***47.8***	***25.2***	13.2	7.1	***4.1***	1.6	99.0
*M*_HIPADD_	Hip adductor	***47.1***	***30.5***	12.6	***4.9***	3.1		98.3
*M*_HIPROT_	Hip internal rotator	***66.3***	***20.0***	6.7	4.0	1.8		98.8
*M*_KNEEFLEX_	Knee flexor	***52.0***	36.0	5.1	3.4	2.1		98.7
*M*_KNEEROT_	Knee internal rotation	93.2	***4.5***	***1.2***	0.6			99.6
*M*_KNEEADD_	Knee adduction	***73.9***	***15.8***	6.2	***2.4***			98.3
*M*_ANKLEDF_	Ankle dorsiflexor	79.3	16.0	***2.9***	1.0			99.2
*M*_LUMBAREXT_	Lumbar extensor	***70.0***	***19.6***	7.9	1.5			99.0
*M*_LUMBARBEND_	Lumbar bending	***60.5***	28.4	***8.4***	1.7			99.0
*M*_LUMBARROT_	Lumbar internal rotator	***51.2***	34.2	***10.9***	***2.7***	0.7		99.7

Principal components retained after Weiss-Indurkhya independent feature selection are shown in bold italic text. Those subsequently retained after Sequential Feature Selection are shown with additional bold italic underline. Rows: Biomechanical variables input into the feature selection pipeline: *F*, muscle forces; *θ*, joint angles; and *M*, joint moments. Columns: Variance explained by individual principal components up to the 6th principal component for each variable. PC abbreviates the term “principal component”, and the numeric suffix is the number of that principal component, e.g. read PC3 as “*third principle component*”

**Table 2:** Group means and standard deviations of the principal component scores for 10 main features that best distinguish between ACLR and control groups during the landing phase of a single-leg forward hop.

Feature	ACLR	Control	*P*	*g*
*F*_HAMS_ PC1	0.62 (2.58)	− 1.25 (1.4)	***< 0.001***	**0.830**
*F*_RF_ PC1	− 1.63 (4.45)	3.31 (5.44)	***< 0.001***	**− 1.027**
*F*_SOL_ PC1	− 1.94 (12.21)	3.79 (11.69)	***< 0.001***	− 0.475
*θ*_HIPROT_ PC2	− 1.70 (22.37)	3.8 (21.19)	0.013	− 0.250
*θ*_KNEEFLEX_ PC1	− 11.32 (83.29)	27.08 (67.92)	***< 0.001***	− 0.488
*θ*_KNEEFLEX_ PC3	− 2.09 (9.82)	3.4 (9.93)	***< 0.001***	− 0.556
*θ*_KNEEROT_ PC3	− 0.99 (11.36)	2.2 (9.04)	0.003	− 0.299
*M*_KNEEROT_ PC3	− 0.11 (1.48)	0.22 (1.65)	0.031	− 0.216
*M*_KNEEADD_ PC1	5.52 (17.32)	− 12.06 (20.25)	***< 0.001***	**0.956**
*M*_LUMBARROT_ PC3	0.27 (2.61)	− 0.59 (2.47)	***< 0.001***	0.334

Principal component scores presented as mean (standard deviation). *P*-values calculated using *t*-test between ACLR and controls at an *a priori* significance level of $\alpha =0.001$ as the 46 features input into Sequential Feature Selection were already significant at $t\ge 2.0$ (approx. $\alpha =0.046)$. Significant *P*-values are presented in bold italic. Effect sizes were calculated using Hedges *g*, with strong effects ($\left|g\right|\ge 0.8$) shown in bold

For each main feature, we qualitatively interpreted the influence of that feature (e.g., *θ*_KNEEFLEX_ PC1) on the original variable waveform (e.g., *θ*_KNEEFLEX_), and described between-group differences, by: (1) plotting the waveforms of the original pooled data (ACLR and controls combined) corresponding to the upper and lower quartiles of the principal component scores (Figs. 1, 2, and 3, top row); (2) plotting the shape of the eigenvector (i.e, the waveform of the principal component coefficients), and its squared correlation with the waveforms of the original data (i.e. variance explained by the feature, which naturally corresponds better with the upper quartile of the original pooled data) (Figs. 1, 2, and 3, bottom row); and (3) finally, we identified whether the ACLR group was “more like” the upper or the lower quartile based on the magnitude and sign of the mean principal component score compared to controls (Table 2). As this is a lengthy and exhaustive procedure, we provide a detailed step-by-step narrative description of the above process for each main feature as Supplementary Material, with the only summary of findings reported here (Table 3).

**Table 3:** Qualitative interpretation of the effect of each principal component in the main feature set, and the corresponding differences between ACLR and control groups.

Feature	Qualitative interpretation*: The principal component…	ACLR group†: Nearest quartile	Effect of principal component‡: The ACLR group tends to have…
*F*_HAMS_ PC1	…upscales the hamstrings force throughout middle of the landing phase, predominantly near the peak	Upper	…greater peak hamstrings force, greater hamstrings force throughout the middle of landing
*F*_RF_ PC1	…upscales the rectus femoris force throughout middle of the landing phase, predominantly near the peak	Lower	…diminished peak rectus femoris force, smaller rectus femoris force throughout the middle of landing
*F*_SOL_ PC1	…upscales the soleus force throughout middle of the landing phase, predominantly near the peak	Lower	…diminished peak soleus force, smaller soleus force throughout the middle of landing
*θ*_HIPROT_ PC2	…increases hip internal rotation angle at foot strike, i.e. hip has greater range of motion through landing	Lower	…less range of motion throughout the landing phase
*θ*_KNEEFLEX_ PC1	…downscales the waveform towards zero, reducing knee flexion angle, particularly after foot strike, i.e. favours straighter knee throughout landing	Lower	…a straighter knee throughout landing phase, predominantly after foot strike
*θ*_KNEEFLEX_ PC3	…reduces instantaneous knee flexion angle at foot strike, i.e. at the instant of footstrike, tends to land with straighter knee initially	Lower	…a straighter knee at the instant of foot strike
*θ*_KNEEROT_ PC3	…downscales the amplitude of oscillatory components knee rotation angle waveform	Lower	…greater oscillations in knee rotation angle waveform, a more internally-rotated knee around mid-phase
*M*_KNEEROT_ PC3	… applies a small modulation to the magnitude and timing of first peak of knee rotation moment waveform	Lower	…more pronounced oscillations in knee rotation moment waveform, out of phase relative to controls
*M*_KNEEADD_ PC1	…downscales the knee adduction moment towards zero throughout the second half of the landing phase, predominantly near the peak	Upper	…lower peak knee abduction moment, lower knee abduction moment throughout the middle of landing
*M*_LUMBARROT_ PC3	…downscales the amplitude and frequency of oscillatory components of lumbar rotator moment waveform	Upper	…less pronounced oscillations in lumbar rotator moment waveform, fewer peaks

^*^Qualitative interpretation: statement describing the meaning of the principal component with respect to its associated variable, i.e. description of the what the principal component does to the overall waveform of that variable. This is determined by analysing the shapes and magnitudes of waveforms of the upper and lower quartiles of the pooled data for that principal component, and comparing them against the waveforms of the principal component coefficient and the percentage of variance explained (Figs. 1, 2, and 3)

^†^ACLR group: indicates whether the mean principal component score for the ACLR group is nearer the upper or lower quartile of principal component scores for the pooled data, based on the principal component scores for each group (Table 2

^‡^Effect of principal component: a statement describing how the principal component impacts the overall waveform for that variable in the ACLR group compared to the control group, based on: (1) the qualitative interpretation of the principal component; and (2) the quartile of the principal component scores that is nearest the average score for the ACLR group

All three principal components of muscle forces, *F*_HAMS_ PC1 (read as *“the first principal component of the hamstrings force”)*, *F*_RF_ PC1 and *F*_SOL_ PC1, differed between ACLR and controls at significance level $\alpha =0.001$ (Table 2). They were interpreted to upscale their respective waveforms (i.e., increase the waveform amplitude), through middle of the landing phase, acting predominantly near their respective waveform peaks (Fig. 1; Table 3). Thus, these features influenced greater peak hamstrings force, but lower peak rectus femoris and soleus forces in the ACLR group.

Two of the four principal components of joint angles, *θ*_KNEEFLEX_ PC1 and *θ*_KNEEFLEX_ PC3, differed between groups at significance level $\alpha =0.001$ (Table 2). *θ*_KNEEFLEX_ PC1 tended to downscale, i.e., reduce the amplitude, of the knee flexion angle waveform throughout landing, while *θ*_KNEEFLEX_ PC3 further reduced the knee flexion angle near foot strike (Fig. 2; Table 3). These features influenced a “straighter” a knee landing strategy in the ACLR group.

Two of the three principal components of joint moments, *M*_KNEEADD_ PC1 and *M*_LUMBARROT_ PC3, differed significantly between ACLR and controls at significance level $\alpha =0.001$ (Table 2). *M*_KNEEADD_ PC1 tended to downscale the knee adduction moment waveform particularly near the peaks in the second half of the landing phase (Fig. 3; Table 3). The ACLR tended to land with less knee *abduction* moment compared to controls. *M*_LUMBARROT_ PC3 tended to downscale both the amplitude and frequency of the lumbar rotator moment waveform, with the ACLR group having reduced range and frequency of lumbar rotator moments (Fig. 3; Table 3).

Eight associated features were found that correlated moderately or strongly with the main features at significance level $\alpha =0.05$, and were also able to distinguish between ACLR and control groups at significance level $t\ge 2.0$ (approx. $\alpha =0.046)$ (Table 4), with six able to distinguish between groups at significance level $\alpha =0.001$.

**Table 4:** Associated features for each main feature in the final set.

Main feature	Associated feature	*ρ*	Mean principal component scores			
			ACLR	Control	*P*	*g*
*F*_HAMS_ PC1	None					
*F*_RF_ PC1	*M*_KNEEFLEX_ PC1	0.638	7.67	− 15.23	***< 0.001***	**1.084**
*F*_SOL_ PC1	*M*_ANKLEDF_ PC1	***− 0.862***	3.09	− 5.96	**< *0.001***	0.347
*θ*_HIPROT_ PC2	None					
*θ*_KNEEFLEX_ PC1	*F*_VAS_ PC1	− 0.536	− 2.52	5.45	***< 0.001***	− 0.712
	*M*_HIPROT_ PC2	− 0.524	0.55	− 0.83	0.002	0.311
	*M*_KNEEADD_ PC2	0.577	− 1.24	1.93	***< 0.001***	− 0.347
*θ*_KNEEFLEX_ PC3	None					
*θ*_KNEEROT_ PC3	None					
*M*_KNEEROT_ PC3	None					
*M*_KNEEADD_ PC1	*θ*_KNEEROT_ PC1	− 0.618	− 7.57	19.66	***< 0.001***	− 0.484
	*M*_HIPROT_ PC1	− 0.611	− 2.33	4.98	***< 0.001***	**− 0.989**
*M*_LUMBARROT_ PC3	*M*_LUMBARBEND_ PC3	0.510	0.49	− 1.21	0.002	0.304

Only associated features with moderate, $0.5\le \left|\rho \right|<0.7$, or strong. $\left|\rho \right|\ge 0.7$, correlation with their main feature, and which were able to discriminate between ACLR and control groups at significance level $t\ge 2.0$ (approx. $\alpha =0.046)$ are shown. Associated features with strong correlation are presented in bold italic with bold italic underline. All correlations are significant at $\alpha =0.05$. *P*-values indicating significant between-group differences in principal component scores at level $\alpha =0.001$ are presented in bold italic. Effect sizes for principal component scores were calculated using Hedges *g*, with strong, $\left|g\right|\ge 0.8$, effects shown in bold

## Discussion

Using automated pattern recognition methods, we found altered biomechanics in individuals 12-24 months after ACLR during the landing phase of a sub-maximal single-leg forward hop. We systematically identified and described 10 main features that distinguished between the landing biomechanics of ACLR and control groups—specifically, 3 principal components of muscle forces, 4 of joint angles and 3 of joint moments, and 8 additional associated features—which advance and reinforce current understanding of altered biomechanics after ACLR. Furthermore, the ability to analyse full temporal waveforms rather than just discrete points, and also to embody a large suite of variables within a small set of main features, are two major advantages of using pattern recognition methods over traditional multivariate analyses. Our hypothesis was supported, as two principal components of knee flexion angle were included as main features, and knee flexor moment was included as an associated feature of rectus femoris force.

Our pattern recognition method systematically detected large between-group differences in landing biomechanics, as evidenced by a number of high principal components (PC1, and some PC2) of variables in our main and associated feature sets, with the PC1 features accounting for between 52.0% and 93.3% of the variance in their respective variables. In particular, the ACLR group demonstrated considerably altered knee-spanning muscle forces and sagittal-plane knee-joint biomechanics. Similar to reported findings for single-legged^9,25,28,45^ and double-legged^20^ landing tasks, individuals with ACLR landed with a more extended knee (*θ*_KNEEFLEX_ PC1) and reduced knee extensor moments (*M*_KNEEFLEX_ PC1 *via* main feature *F*_RF_ PC1). This latter finding manifested as diminished quadriceps forces in the ACLR group (*F*_RF_ PC1, and *F*_VAS_ PC1 *via* main feature *θ*_KNEEFLEX_ PC1). Together with greater hamstrings forces (*F*_HAMS_ PC1) in the ACLR group, this indicated elevated hamstrings-quadriceps co-contraction throughout landing, a finding frequently reported in electromyographic studies in individuals with ACLR.^42^ Furthermore, these results could imply lower patellofemoral-joint loading in the ACLR group, which is associated with risk of future osteoarthritis.^39,40^ Our results are in agreement with those commonly reported for biomechanical alterations after ACLR surgery^12,15,25,27,45^ and our method could identify them with an exceptionally high degree of certainty (at the 99% confidence level) providing confidence in the validity of our approach.

However, our finding of reduced ankle plantar flexor function (*F*_SOL_ PC1, and its associated feature *M*_ANKLEDF_ PC1) differs from previously reported results for landing activities, which found greater hip extension and ankle plantar flexor moments in the ACLR limb relative to the uninjured contralateral limb^9,28^ during such tasks, typically described as a redistribution of torque away from the compromised knee.^38^ In our present study, reduced ankle plantar flexor function with reduced knee (and hip; not selected as a main or associated feature; see Supplementary Material) extensor moments indicate a global reduction in kinetic demand across the ACLR limb. This could occur as a result of the straighter, stiffer landing strategy adopted by individuals with ACLR, which we have shown provides the same centre-of-mass modulation with less muscular effort, but at the expense of greater axial knee-joint loading.^38^ Our results may be explained by the reduced demands of our constrained sub-maximal single-leg forward hops, compared to the maximum-effort hops of Gokeler *et al*.,^9^ for example.

We identified very subtle differences in the temporal waveforms of the biomechanical variables, and could say with at least 95% certainty that these were not simply due to measurement noise. This was evidenced by the inclusion of low principal components (PC3, and some PC2) in our main and associated feature sets, with PC3 features accounting for only 1.2% to 10.9% of the variance in those respective variables (Table 1). These small between-group differences could not be discerned by comparing group mean waveforms of the original data alone (Supplementary Material). Our results suggest that these subtle aberrations in non-sagittal-plane angles and moments may, in fact, be salient features of ACLR biomechanics 12-24 months post-surgery, that may not be easily detectable in a clinical setting. Specifically, these subtle biomechanical aberrations were indicated by the inclusion of low principal components of: (1) the knee (*θ*_KNEEFLEX_ PC3, *θ*_KNEEROT_ PC3 and *M*_KNEEROT_ PC3), each of which accounted for less than 3% of the variance in their respective variables; (2) the hip (*θ*_HIPROT_ PC2), accounting for 8.9% of the variance of hip rotation angle; and (3) the back (*M*_LUMBARROT_ PC3, *M*_LUMBARBEND_ PC3), each of which accounted for less than 11% of the variance in their respective variables (Table 1). Both *θ*_KNEEFLEX_ PC3 and *θ*_HIPROT_ PC2 tended to perturb their respective joint angles at foot-strike, while the others modulated oscillations in the temporal waveforms of their respective variables (Figs. 1, 2, and 3; Table 3), suggesting the ACLR group landed with a greater level of frontal- and transverse-plane unsteadiness or instability. Allowing individuals after ACLR to return to sport without specifically identifying and addressing these subtle instabilities may cause individuals to retain them as they increase the intensity and volume of their physical activity^22^, elevating risk of re-injury and future osteoarthritis. Currently, detecting these small aberrations reliably and robustly is challenging in a clinical setting, however, future technological advancements may enable such analyses to be undertaken routinely.

Overall, non-sagittal-plane variables, specifically those associated with hip rotation, knee rotation and knee adduction angles and moments, were strongly represented in the main and associated feature sets (Tables 3 and 4). Most notably, our results add new evidence supporting the importance of abnormal/deficient coordination of hip rotation in biomechanics of ACLR individuals.^17,20,25^ In the ACLR group, we found diminished internal hip rotator moments (*M*_HIPROT_ PC1 and *M*_HIPROT_ PC2) (Table 4), as well as a tendency for reduced range of hip rotation angle during landing (*θ*_HIPROT_ PC2). Holistically, these findings suggest that reduced hip rotator muscle function in the ACLR group is associated with a more extended knee (*θ*_KNEEFLEX_ PC1), less internal rotation (*θ*_KNEEROT_ PC1), and diminished internal knee abduction moments (*M*_KNEEADD_ PC1) (Tables 3 and 4). Thus, our results reinforce the role of dynamic coupling in hop-landing biomechanics,^38^ and the need to consider the entire kinetic chain in the assessment and rehabilitation of individuals after ACLR.

Furthermore, deficiencies in hip external rotator strength can predict single-leg hop performance in ACLR individuals 8 months post-operatively,^17^ and, together with deficient hip abductor muscle function, is a risk factor for lower-limb injuries during sports.^19^ Although hip external rotator muscles, such as gluteus medius, were not selected as either main or associated features, their deficient action could be inferred from *M*_HIPROT_ PC1, *M*_HIPROT_ PC2 and *θ*_HIPROT_ PC2, and confirmed by examining the actual group mean waveforms for gluteus medius (Supplementary Material). Additionally, it is possible that many individuals in our present ACLR cohort may have been at some risk of re-injury at the time of testing, as deficient hip rotation kinetics strongly predicted ACL re-injury after returning to sport.^30^

Our present study is not without limitations. Firstly, PCA allows for examination of bimodal data (trial vs time, or variable vs time), whereas motion data is trimodal (trial vs variable vs time).^11^ A multimodal analysis method, such as Parallel Factor Analysis (PARAFAC), could potentially simultaneously identify the components describing individual or group differences in motion patterns, determine the instants when components are influential, and examine relationships between variables.^11^ However, the PCA calculation is a convex problem with unique solutions, while PARAFAC solves a non-convex problem that must, during its execution, examine locally-optimal solutions. Thus, overall PARAFAC is computationally more expensive than PCA, but not necessarily more accurate.

Secondly, SFS is a suboptimal method for feature selection. It may be possible to find a “better” set of discriminating features. However, feature selection problems may not be solved optimally in polynomial time^37^; therefore, only suboptimal methods such as SFS are feasible in practice.^14^ This implies that SFS, with our selection threshold of 10 features, could retain a few “weak” features while discarding some “stronger” ones. Yet, our use of *t*-tests at approximately 95% significance level as a preliminary reduction method guarantees that all remaining features are statistically-relevant.

Thirdly, the present set of results is valid only for distinguishing between groups using data from single-leg hop landings. We chose to examine single-leg hop landings as they are an important task used the clinical assessment of ACLR individuals for return-to-sport. Nevertheless, our methodology is not task-specific. Inputting biomechanical data from other tasks, such as running or squatting, may reveal contrasting sets of discriminating features, which may be used to assess individuals’ performances in those tasks. Future studies may pool data from many tasks to reveal common discriminating features.

Finally, our calculation of muscles forces did not consider subject-specific muscle activation patterns. We used CMC^41^ in OpenSim to calculate muscle activations, which can be constrained by inputting normalised electromyographic signals for one or more muscles if desired. However, electromyographic data were not collected in this study as it would have exacerbated the long testing time (>3 h) and participant fatigue. Nevertheless, our use of CMC, which combines static optimisation, forward dynamics and feedback control to calculate muscle activations, improves on previous estimates using static optimisation alone.^39,40^

In conclusion, we found altered landing biomechanics 12–24 months after ACLR using an automated pattern recognition approach. Our findings reinforce the importance of non-sagittal-plane biomechanical variables, as well as subtle aberrations, in clinical assessments after ACLR. Automated methods for classifying individuals with ACLR may, in future, have application in machine learning-based software tools for improving clinical decision-making around the time of return-to-sport.

## Supplementary Information

Below is the link to the electronic supplementary material.

Supplementary file1 (PDF 660 kb).
