# Supplementary material for: Biomechanical Markers of Forward Hop-Landing After ACL-Reconstruction: A Pattern Recognition Approach
Source: Ann Biomed Eng. 2022 Jan 31;50(3):330–42. doi: 10.1007/s10439-022-02921-4 (PMC8847210; doi:10.1007/s10439-022-02921-4)

SUPPLEMENTARY MATERIAL

BIOMECHANICAL MARKERS OF FORWARD HOP-LANDING AFTER ACL-RECONSTRUCTION: A PATTERN RECOGNITION APPROACH

Prasanna Sritharan[1]*, Mario A. Muñoz[2]*, Peter Pivonka[3], Adam L. Bryant[4], Hossein Mokhtarzadeh[5], Luke G. Perraton[6]

co-first authors

[1]La Trobe Sports & Exercise Medicine Research Centre, La Trobe University, Victoria, Australia

[2]School of Mathematics & Statistics, University of Melbourne, Victoria, Australia

[3]School of Mechanical, Medical & Process Engineering, Queensland University of Technology, Queensland, Australia

[4]Centre for Health, Exercise and Sports Medicine, University of Melbourne, Victoria, Australia

[5]School of Engineering, University of Melbourne, Victoria, Australia

[6]Department of Physiotherapy, Monash University, Victoria, Australia

Results: Qualitative interpretation of main features and between-group differences

The variance explained by each of the three principal components of muscle forces, F_{HAMS} PC1 (read as *"the first principal component of the hamstrings force")*, F_{RF} PC1 and F_{SOL} PC1, was greatest through the middle of the landing phase, reaching a maximum near the peak values of the respective principal components (Figure 1, bottom row). Thus, all three principal components were interpreted to upscale their respective waveforms, i.e. increase the waveform amplitude, through middle of the landing phase, acting predominantly near their respective waveform peaks (Table 3). The scores for these principal components, F_{HAMS} PC1, F_{RF} PC1 and F_{SOL} PC1, differed significantly between ACLR and controls, with strong effect sizes for F_{HAMS} PC1 and F_{RF} PC1 (Table 2). The principal component scores for the ACLR group were greater than controls for F_{HAMS} PC1, but smaller than controls for F_{RF} PC1 and F_{SOL} PC1 (Table 2). Thus, for F_{HAMS} PC1, the average waveform of the hamstrings force for the ACLR group was closer to the waveform of the pooled data that corresponded to the upper quartile of principal scores; and for each of F_{RF} PC1 and F_{SOL} PC1, the average waveform of the hamstrings force for the ACLR group was closer to the waveform of the pooled data that corresponded to the lower quartile of principal scores for the respective features (Table 2; and Figure 1, top row). Thus, the ACLR group tended to have greater peak hamstrings force, but lower peak rectus femoris and soleus forces.

Of the four principal components of joint angles in the final feature set, only two, θ_{KNEEFLEX} PC1 and θ_{KNEEFLEX} PC3, differed between ACLR and controls at significance level $\alpha = 0.001$ (Table 2). For both of these features, the principal component score for the ACLR group was greater than controls (Table 2), indicating that the average waveform of the knee flexion angle for the ACLR group was closer to the waveform of the pooled data that corresponded to the upper quartile of principal scores for that feature (Figure 2, top row). θ_{KNEEFLEX} PC1 tended to downscale the knee flexion angle waveform towards zero, i.e. reduce

the amplitude of the waveform, and explained over 50% of the variance throughout landing, reaching over 90% from the middle to the end of the phase (Figure 2, bottom row). $\theta_{\mathrm{KNEEFLEX}}$ PC3 tended to reduce the initial value for the knee flexion angle near foot strike, with the variance explained rapidly diminishing from almost 40% to near zero after that instant (Figure 2, bottom row). Together, $\theta_{\mathrm{KNEEFLEX}}$ PC1 and $\theta_{\mathrm{KNEEFLEX}}$ PC3 indicated that the ACLR group tended to land with a less knee flexion at foot strike, and then maintain a lower knee flexion angle through to the end of landing. Therefore, overall, the ACLR group landed with a "straighter" knee compared to controls (Table 3).

Two of the three principal components of joint moments in the final feature set, M_{KNEEADD} PC1 and $M_{\mathrm{LUMBARROT}}$ PC3, differed significantly between ACLR and controls at significance level $\alpha = 0.001$, with a strong effect size for M_{KNEEADD} PC1 (Table 2). For both of these features, the average principal component score was greater for the ACLR group was greater than controls (Table 2), indicating that the average waveforms of the knee adduction moment and lumbar rotation moment for the ACLR group were both closer to the waveforms of the pooled data that corresponded to the upper quartiles of principal scores for those respective features (Figure 3, top row). M_{KNEEADD} PC1 acted predominately in the second half of the landing phase, explaining over 80% of the variance near the waveform peak, and tended to downscale the knee adduction moment waveform towards zero, i.e. reduce the amplitude of the waveform. As the sign of the knee adduction moment waveforms were negative throughout landing, M_{KNEEADD} PC1 indicates that the ACLR group landing with less knee *abduction* moment, particularly in the second half of the landing phase. $M_{\mathrm{LUMBARROT}}$ PC3 tended to downscale both the amplitude and frequency of the lumbar rotation moment waveform, indicating that the ACLR group applied smaller lumbar rotation moments with fewer peaks.

Figure S1. Mean waveforms of muscle forces for ACLR (red) and control (blue) groups, and both groups combined (black), during the landing phase of the single-leg forward hop.

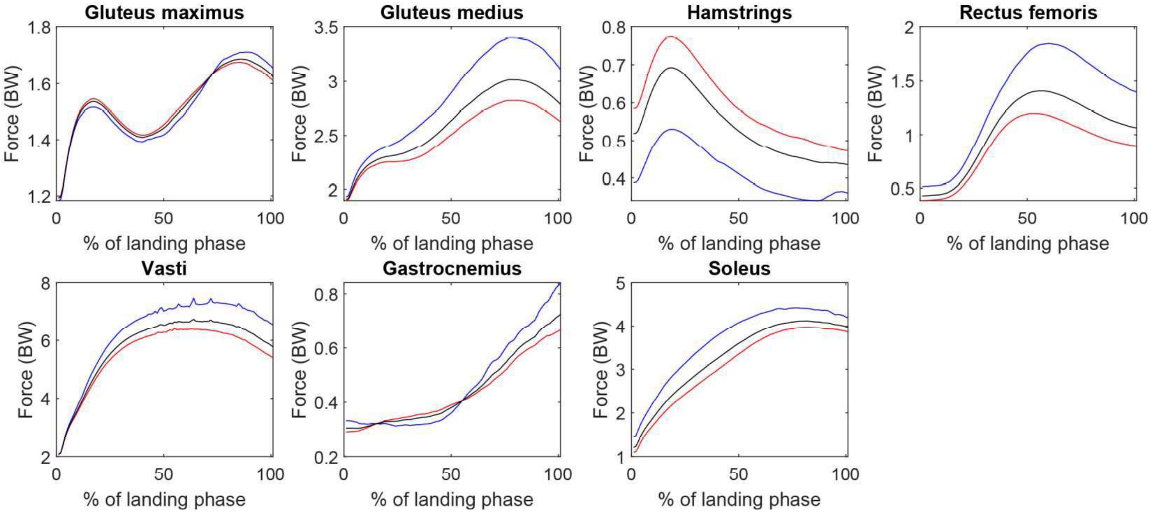

Figure S2. Mean waveforms of joint angles for ACLR (red) and control (blue) groups, and both groups combined (black), during the landing phase of the single-leg forward hop.

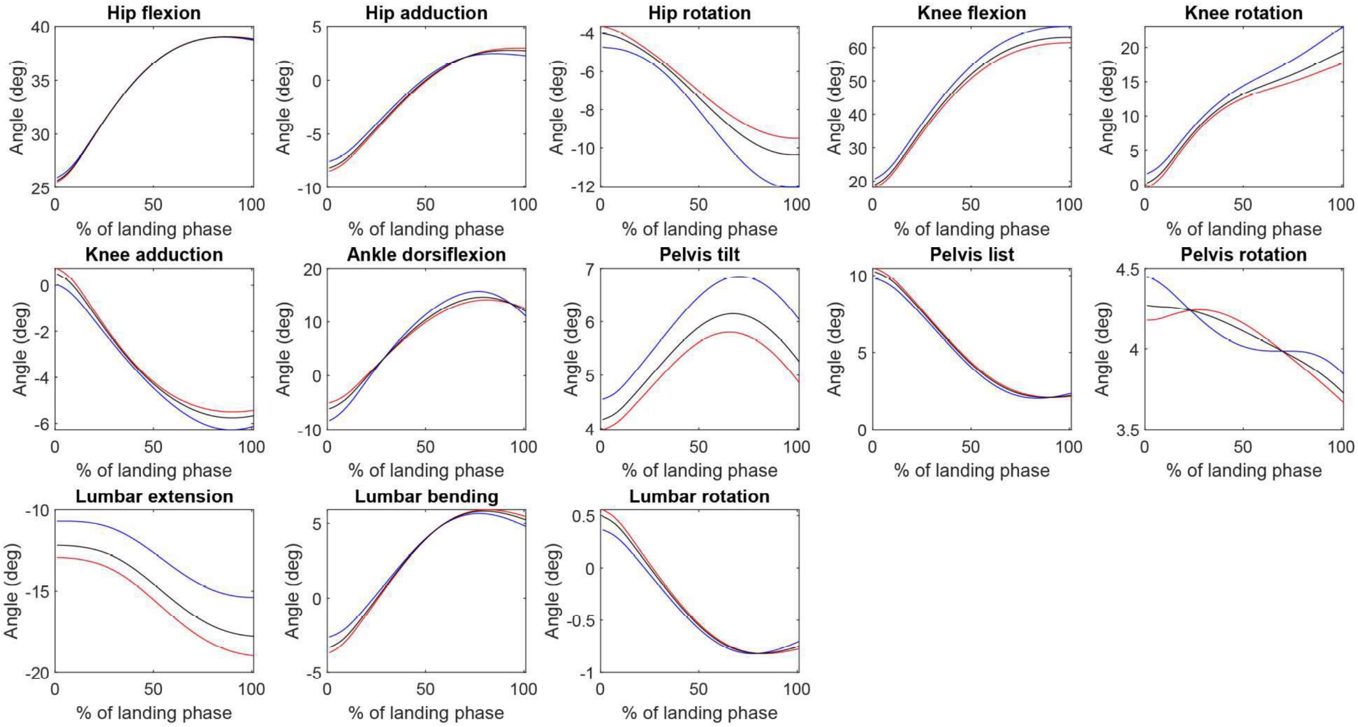

Figure S3. Mean waveforms of joint moments for ACLR (red) and control (blue) groups, and both groups combined (black), during the landing phase of the single-leg forward hop.

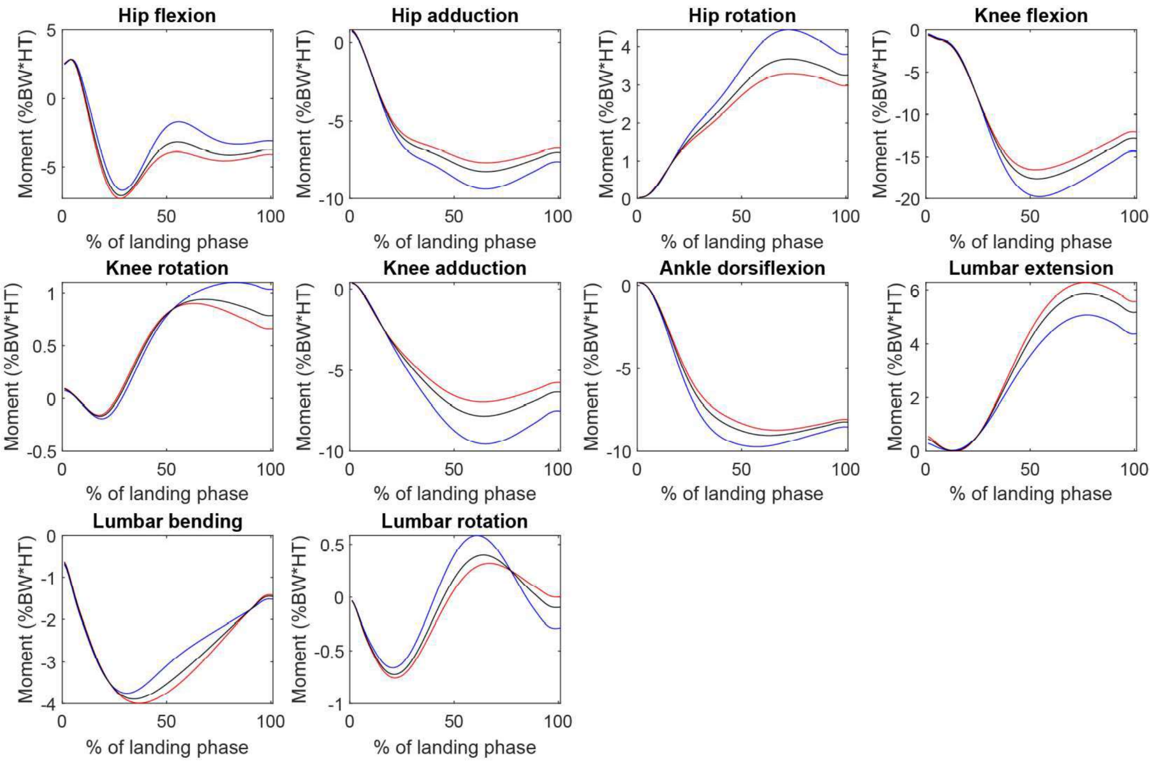

Supplement: Supplementary file 1 — Supplementary file1 (PDF 660 kb). [file 10439_2022_2921_MOESM1_ESM.pdf]
